# Local Ultrasound-Facilitated Thrombolysis in High-Risk Pulmonary Embolism: First Dutch Experience

**DOI:** 10.1007/s00270-019-02200-1

**Published:** 2019-03-12

**Authors:** Maria A. de Winter, Einar A. Hart, Daniel A. F. van den Heuvel, Adriaan Moelker, Rutger J. Lely, Karin A. H. Kaasjager, Pieter R. Stella, Steven A. J. Chamuleau, Adriaan O. Kraaijeveld, Mathilde Nijkeuter

**Affiliations:** 1 Department of Internal Medicine, University Medical Center Utrecht, Heidelberglaan 100, Utrecht, The Netherlands; 2 Department of Cardiology, University Medical Center Utrecht, Heidelberglaan 100, Utrecht, The Netherlands; 3 Department of Interventional Radiology, St. Antonius Hospital, Koekoekslaan 1, Nieuwegein, The Netherlands; 4 Department of Radiology and Nuclear Medicine, Erasmus Medical Center Rotterdam, Doctor Molewaterplein 40, Rotterdam, The Netherlands; 5 Department of Interventional Radiology, VU University Medical Center, De Boelelaan 1117, Amsterdam, The Netherlands

**Keywords:** Pulmonary embolism, Thrombolytic therapy, Emergency treatment

## Abstract

**Purpose:**

To provide insight into the current use and results of ultrasound-facilitated catheter-directed thrombolysis (USAT) in patients with high-risk pulmonary embolism (PE).

**Introduction:**

Systemic thrombolysis is an effective treatment for hemodynamically unstable, high-risk PE, but is associated with bleeding complications. USAT is thought to reduce bleeding and is therefore advocated in patients with high-risk PE and contraindications for systemic thrombolysis.

**Methods:**

We conducted a retrospective cohort study of all patients who underwent USAT for high-risk PE in the Netherlands from 2010 to 2017. Characteristics and outcomes were analyzed. Primary outcomes were major (including intracranial and fatal) bleeding and all-cause mortality after 1 month. Secondary outcomes were all-cause mortality and recurrent venous thromboembolism within 3 months.

**Results:**

33 patients underwent USAT for high-risk PE. Major bleeding occurred in 12 patients (36%, 95% CI 22–53), including 1 intracranial and 3 fatal bleeding. All-cause mortality after 1 month was 48% (16/33, 95% CI 31–66). All-cause mortality after 3 months was 50% (16/32, 95% CI 34–66), recurrent venous thromboembolism occurred in 1 patient (1/32, 3%, 95% CI 1–16).

**Conclusions:**

This study was the first to describe characteristics and outcomes after USAT in a study population of patients with high-risk PE only, an understudied population. Although USAT is considered a relatively safe treatment option, our results illustrate that at least caution is needed in critically ill patients with high-risk PE. Further research in patients with high-risk PE is warranted to guide patient selection.

## Introduction

Pulmonary embolism (PE) is a common cardiovascular disease that can result in significant morbidity and death. PE patients with hemodynamic shock or hypotension are classified as high-risk, those with right ventricular (RV) dysfunction and/or myocardial injury as intermediate-risk and patients without those signs as low risk of mortality [1]. In the high-risk group, comprising 5% of all PE patients, an in-hospital mortality of 25–65% is found, depending on clinical presentation and timely availability of treatment [1–3]. Systemic thrombolysis (ST) is standard of care in high-risk patients [1–4]. It has been shown to restore pulmonary perfusion more rapidly compared to standard anticoagulation alone, thereby improving RV function and reducing mortality [5–8]. However, systemic thrombolysis carries a 20% risk of major bleeding, including a 2–3% risk of intracranial hemorrhage [1, 4, 9, 10]. Consequently, risk factors for bleeding are considered (relative) contraindications for this treatment [8, 11]. Currently, in patients with high-risk PE in whom ST is contraindicated or has failed, surgical embolectomy or ultrasound-assisted catheter-directed thrombolysis (USAT) is advised [1]. USAT, using the EkoSonic Endovascular system (EKOS Corporation; Bothell, WA, USA) is currently the most studied catheter-based technique using a lower dose of thrombolytic agent. With this catheter, ultrasound is used to drive the thrombolytic agent directly into plasminogen receptor sites within the thrombus and separate fibrin strands more efficiently [12, 13]. Previous studies on USAT and other local thrombolytic interventions for PE did show a reduction of pulmonary artery pressure and improved echocardiographic parameters, with a favorable safety profile [14–27]. However, these studies had a short follow-up and lacked clinically relevant outcomes, such as major bleeding. More importantly, study populations were relatively small and involved mainly intermediate-risk patients. Whereas most evidence regarding efficacy and safety of USAT originates from data on intermediate-risk patients, its main indication is for high-risk PE. In previous literature, patients with high-risk PE were either not or scarcely included. Ideally, a randomized controlled trial comparing ST and USAT would be undertaken to identify the best strategy for patients with high-risk PE. However, these patients are by definition hemodynamically unstable and often require immediate life-saving therapy that cannot be postponed by asking informed consent. Therefore, we need to assess the effect of this strategy using data from case series and registries. Currently, USAT is only advised in high-risk patients in whom ST is contraindicated or has failed [1]. However, evidence supporting this strategy is scarce.

The aim of this retrospective study was to provide insight into the use and outcomes of USAT in patients with high-risk PE treated according to current guidelines.

## Materials and Methods

### Study Design and Setting

This retrospective cohort study was conducted at all hospitals performing USAT in the Netherlands, including three academic hospitals (University Medical Center Utrecht, Utrecht, the Netherlands, Erasmus Medical Center, Rotterdam, the Netherlands and VU University Medical Center, Amsterdam, the Netherlands) and one tertiary referral center for cardiovascular and pulmonary disease (St. Antonius hospital, Nieuwegein, the Netherlands).

### Selection of Participants

We included all patients that underwent USAT for PE in the Netherlands since its introduction in 2010 until July 2017. Patients were identified from a database maintained by interventional cardiologists performing USAT, by searching radiology reports on ‘thrombectomy, ‘fibrinolysis’, ‘pulmonary arteries’, ‘thoracic arteries’ and ‘EKOS’ and by using software implemented in the local electronic medical record. Demographic data and clinical information were extracted from medical records.

### Definitions

PE was, according to guidelines, diagnosed with computed tomographic pulmonary angiography (CTPA) or, when considered unsafe, a high clinical suspicion with or without RV dysfunction on echocardiography [1]. High-risk PE was defined as PE with hemodynamic shock or hypotension (systolic blood pressure < 100 mmHg or a decline of > 40 mmHg) [1]. Shock is defined as hypotension or other signs of reduced tissue perfusion (altered mental state, oliguria, clammy and pale skin, hyperlactatemia) [1]. Intermediate-risk PE was defined as PE with RV dysfunction and/or elevated cardiac biomarkers [1]. The presence of RV dysfunction (dilated, enlarged or decompensated right ventricle, right heart strain, RV failure or dysfunction) was extracted from echocardiography and CTPA reports. Bleeding risk factors are based on American College of Chest Physicians and European Society of Cardiology guidelines [1, 28]. Major bleeding risk factors include recent hemorrhagic stroke, surgery, trauma, head injury, gastrointestinal bleeding, central nervous system malignancies and active bleeding. Minor bleeding risk factors are recent transient ischemic attack, current therapeutic anticoagulation, pregnancy, traumatic resuscitation, refractory hypertension, end stage liver disease, infectious endocarditis and active stomach ulcer.

### Intervention

According to guidelines, ST is administered in high-risk PE patients without major contraindications (abovementioned major risk factors) [1]. USAT is considered in patients with high-risk PE and contraindications for ST whose condition is otherwise stable enough to be transported to the catheterization laboratory. Local practice varies regarding combination with other therapies, including extracorporeal membrane oxygenation (ECMO), thrombus aspiration and ST.

USAT was performed by interventional cardiologists (Utrecht) or radiologists (Nieuwegein, Rotterdam, Amsterdam). Venous access was obtained via the femoral or internal jugular vein. The catheters are placed under fluoroscopic guidance according to CTPA images. Before inserting the EKOS-catheters, a pulmonary angiogram was performed. In case of bilateral PE, two EKOS-catheters were placed through the thrombus in the pulmonary artery under fluoroscopy, one on each side, and 12 mg of Alteplase was administered per catheter for 12 h. In case of unilateral PE, one EKOS-catheter was placed, and 24 mg of Alteplase was locally administered for 24 h. Echocardiography is frequently performed after catheter removal to assess the effect of USAT on RV dysfunction. Moreover, the patient’s clinical condition is monitored closely. In case of insufficient clinical and echocardiographic improvement, repeat CTPA is often performed to aid further clinical decisions regarding additional therapies. Heparin was administered according to predefined protocols. In one hospital (Utrecht), an intravenous bolus of heparin of 5000 IE or 80 IE per kilogram body weight is administered before USAT, and continuous infusion is started after USAT with a target activated Partial Thromboplastin Time (aPTT) of 2–2.5. In the other hospitals (Nieuwegein, Rotterdam, Amsterdam) heparin is administered concomitantly, based on a target level of 2.5–3, and continued afterwards. If no bleeding had occurred within 24–48 h after thrombolysis, standard anticoagulation therapy consisting of direct oral anticoagulants (DOAC), low molecular weight heparin (LMWH) or a vitamin K antagonist (VKA) was started.

### Outcome Assessment

Primary outcomes were all-cause mortality during the first month of follow-up as well as major bleeding, including intracranial hemorrhage and fatal bleeding, as defined by the International Society on Thrombosis and Haemostasis (ISTH) [28]. Secondary outcomes were all-cause mortality and recurrent VTE after 3 months of follow-up, objectively confirmed by CTPA, perfusion-scintigraphy, pulmonary angiography, compression ultrasound or phlebography [1]. All outcomes were evaluated using clinical information as noted in patient files.

### Analysis

Baseline characteristics and outcomes are reported as percentages or median with interquartile range (IQR) as variables were non-normally distributed. Patients treated with USAT and other therapies or USAT only were compared using two-sided Chi square and Fisher’s exact tests. All statistical analyses were performed with SPSS version 21 (SPSS Inc., Chicago, Illinois, USA). *p* values < 0.05 were considered significant.

## Results

### Characteristics of Study Subjects

**Fig. 1:**
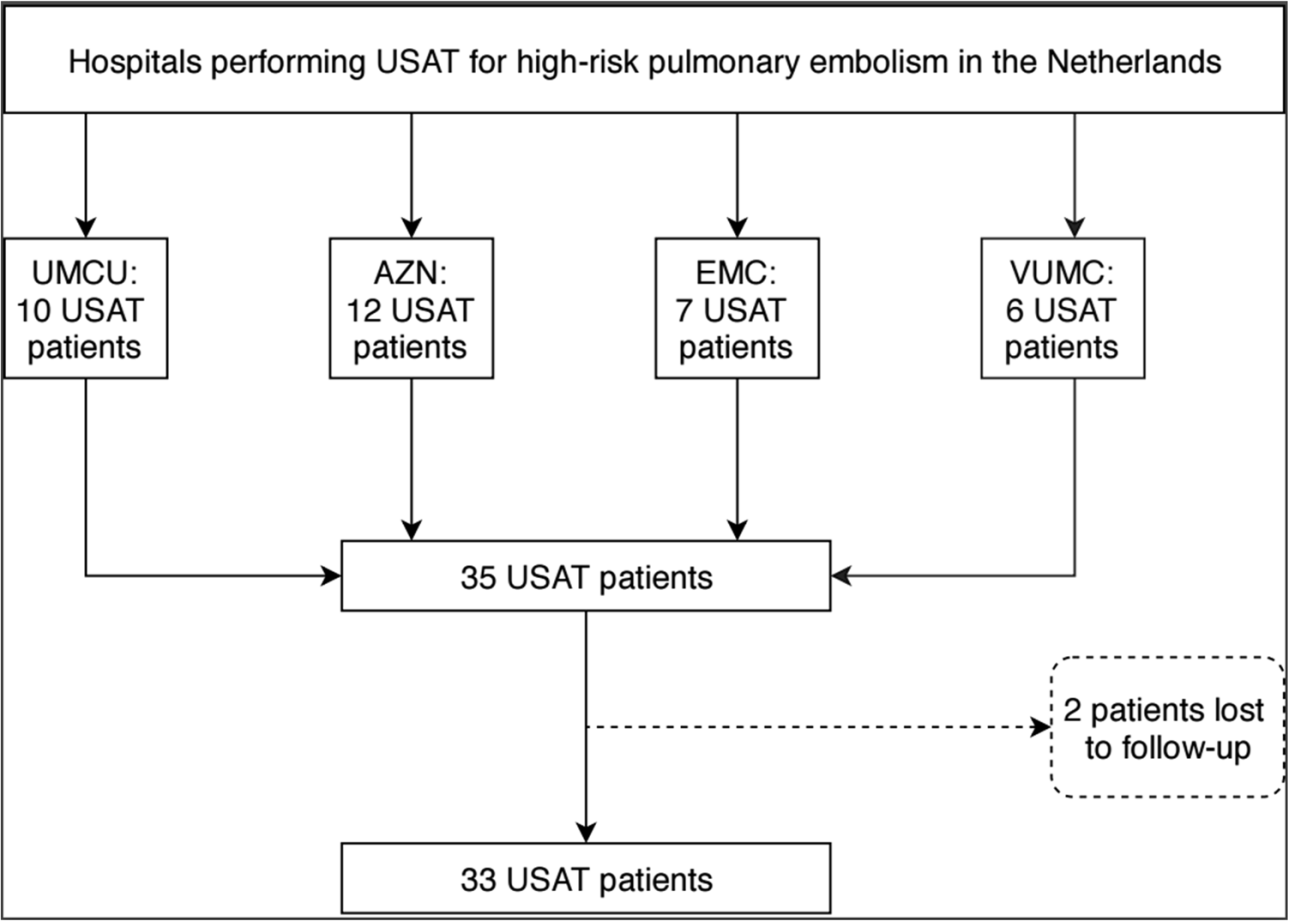
Flowchart of the included patients. *AZN* St. Antonius Hospital Nieuwegein, *EMC* Erasmus Medical Center Rotterdam, *UMCU* University Medical Center Utrecht, *USAT* ultrasound-facilitated, catheter-directed local thrombolysis, *VUMC* VU University Medical Center Amsterdam

We identified 35 patients with high-risk PE that underwent treatment with USAT in the Netherlands (Fig. 1). Two patients were lost to follow-up. Baseline characteristics are shown in Table 1. Before proceeding to therapy, definitive diagnosis was obtained by CTPA in 26/33, and by echocardiography in 5/33 patients. In two patients, PE was suspected on clinical grounds or high pulmonary artery pressures during coronary angiography, and later confirmed by CTPA and pulmonary angiography, respectively. USAT was performed bilaterally in 30/33 patients. Average dose of thrombolytic agent used during USAT in 25 patients treated with Alteplase was 26 mg (SD 11). Three patients received a different thrombolytic agent. USAT was discontinued earlier because of bleeding (*n* = 2) or in case of death during treatment (*n* = 3). In these patients, average dose of Alteplase is unknown.

**Table 1:** Baseline characteristics

	*n* (% of 33)
*Demographic data*	
Age (median years (IQR))	63 (51–71)
Female (%)	17 (52)
*Patient related factors*	
History of VTE	8 (25)^a^
Active malignancy	8 (24)
Therapeutic anticoagulation (VKA, DOAC)	2 (6)
*Clinical status*	
Hypotension (SBP < 100 mmHg)	18 (62)^b^
Shock	31 (93)^a^
RV dysfunction	30 (97)^c^
Resuscitation	20 (61)
Mechanical ventilation	16 (49)^a^
Major bleeding risk factors	
1 risk factor	13 (39)
2 or more risk factors	1 (3)
Minor bleeding risk factors	
1 risk factor	6 (18)
2 or more risk factors	1 (3)
*Indication for USAT*	
Contraindications for ST	23 (70)
Insufficient clinical improvement after ST	6 (18)
Unknown	4 (12)

*DOAC* direct oral anticoagulants, *IQR* interquartile range, *SBP* systolic blood pressure, *USAT* ultrasound-facilitated, catheter-directed local thrombolysis, *ST* systemic thrombolysis, *VKA* vitamin K antagonist

^a^*n* = 32; ^b^*n* = 29; ^c^*n* = 31

### Outcomes

Table 2 shows primary and secondary outcomes. Twelve patients (36%, 95% CI 22–53) suffered from major bleeding, as specified in Table 3. Three patients died from bleeding (3/33, 9%). One of these patients presented with both high-risk PE and ischemic stroke, which was subject to hemorrhagic transformation after USAT. Two other patients with fatal bleeding both experienced traumatic resuscitation resulting in multiple rib fractures and died from hypovolemic shock after severe thoracic bleeding and bleeding from access sites.

**Table 2:** Outcomes

	*n* (% of 33)	95% CI
*Primary outcomes (after 1* *month)*		
Major bleeding	12 (36)	22–53
All-cause mortality	16 (49)	31–66
*Secondary outcomes (after 3* *months)*		
All-cause mortality	16 (50)^a^	34–66
Recurrence of VTE	1 (3)^a^	1–16
*Other outcomes*		
Hospital length of stay (median days (IQR))	17 (10–30)^b^	

*CI* Confidence interval, *IQR* interquartile range, *VTE* venous thrombo-embolism

^a^*n* = 32; ^b^Assessed in all patients surviving the first month of follow-up

**Table 3:** Major bleeding

Major bleeding (as defined by the ISTH^23^)	***n***
Access site hematoma	4
Intrathoracic bleeding (hemothorax or chest wall after traumatic resuscitation or surgery)	4
Intraabdominal hematoma	3
Mucosal bleeding (nasal)	2
Bleeding from ECMO cannula site	1
Hematoma on lower arm, causing compartment syndrome	1
Hemorrhagic transformation of ischemic stroke	1
Total number of major bleeding episodes in 12 patients with major bleeding	16

*ECMO* extracorporeal membrane oxygenation, *ISTH* International Society on Thrombosis and Haemostasis, *USAT* ultrasound-facilitated, catheter-directed local thrombolysis, *VKA* vitamin K antagonist, *VTE* venous thrombo-embolism

Thirteen patients (39%, 95% CI 25–56) died from causes other than bleeding, of whom four did not respond to USAT and one died from recurrent PE. Irreversible brain damage, organ failure, heparin-induced thrombocytopenia, sepsis and glioblastoma led to the death of the other patients. No other episodes of recurrent VTE were observed.

### USAT and Additional Therapies

Table 4 illustrates treatment and outcomes of fourteen patients that received additional therapy. Thrombus aspiration was performed concomitant with USAT in four patients. In the other cases, when the initial treatment of either ST or USAT led to insufficient improvement, other strategies were opted for.

**Table 4:** Additional therapies in USAT patients

Patient	ST	Thrombus aspiration	VCF	ECMO	Major bleeding	Mortality
1	Full dose	–	Yes	–	–	Yes
2	–	Yes	Yes	–	–	–
3	Full dose	Yes	–	Yes	–	Yes
4	Low dose	Yes	–	–	Yes	Yes
5	Loading dose	Yes	–	–	–	Yes
6	Full dose	Yes	–	–	Yes	–
7	–	Yes	–	Yes	–	–
8	–	Yes	–	–	Yes	–
9	–	Yes	–	–	–	–
10	Lower dose	–	–	–	–	Yes
11	Lower dose	–	–	–	–	Yes
12	Lower dose	–	–	Yes	–	–
13	Lower dose	–	–	–	–	–
14	Full dose	–	–	Yes	Yes	Yes
Total	10	8	2	4		

*ECMO* extracorporeal membrane oxygenation, *ST* systemic thrombolysis, *USAT* ultrasound-facilitated, catheter-directed local thrombolysis, *VCF* vena cava filter

Of fourteen patients treated with USAT in combination with ST or thrombus aspiration, four patients suffered from major bleeding (29%, 95% CI 12–55), including one fatal. In total, seven patients died (50%, 95% CI 27–73). Similarly, in the group of patients treated with USAT only (*n* = 19, of whom two with ECMO support), major bleeding occurred in 42% (95% CI 23–64), whereas death occurred in 47% (95% CI 27–28). A somewhat higher mortality was seen in patients treated with both USAT and ST (*n* = 10) compared to patients treated with USAT with or without thrombus aspiration (*n* = 23) (70 vs. 39%, *p* = 0.14). The incidence of major bleeding was similar between those groups (30 vs. 39%, *p* = 0.71).

## Discussion

The aim of this study was to provide insight into current use and outcomes of USAT for high-risk PE in the Netherlands. We observed a very high rate of major bleeding and a high mortality rate. According to guidelines, USAT is to be considered in patients with high-risk PE and relative contraindications to ST. Our results indeed indicate a critically ill high-risk population, reflecting a real-world clinical setting. This case series adds important data on the characteristics and outcomes of this understudied patient population.

In our study population, major bleeding occurred in 36%, of which a quarter fatal. Several reasons can be identified for this higher incidence compared to ± 10% major bleeding in previous literature. Our study population involved patients with high-risk PE only, as opposed to 12–22% with high-risk PE in other studies [15, 16, 18, 19, 24, 25]. Furthermore, our findings are based on real-world data and reflect how current guidelines are put into practice. This means that our study population involves a large proportion of patients with contraindications to ST (in particular recent surgery or traumatic resuscitation) or whose condition did not improve despite ST. Finally, a high percentage in our cohort had major bleeding risk factors. All patients suffering from fatal bleeding after USAT had at least one minor or major risk factor. This restates that, especially in the presence of major risk factors, USAT carries a serious risk of major bleeding [4, 7].

A higher all-cause mortality was observed in our population compared to other studies [18, 19, 24, 25]. This could, similarly, reflect our study population of high-risk patients only, whose risk of death is almost ten times that of those with intermediate-risk PE [1, 4]. However, a recent meta-analysis on catheter-directed therapies for PE found an all-cause mortality of 13% in a separately analyzed group of 186 patients with massive (high-risk) PE [16]. This could be explained by differences in indication for USAT and patient selection. Our population consisted of those at highest risk among high-risk patients, a subgroup presumably not included in previous literature. A higher mortality was observed in patients who received USAT and ST concurrently. The incidences of major and fatal bleeding were similar, which may be explained by severe PE or comorbidities, as ST was administered in case of persistent or recurrent hemodynamic instability after USAT or vice versa, indicating a severely high-risk situation.

The population of patients with high-risk PE is very heterogeneous, ranging from mildly hypotensive, conscious patients to patients in cardiac arrest in which everything must be brought into play. Current guidelines aimed at high-risk PE patients should apply to both, while actual clinical management clearly differs between patients. Treatment decisions can be particularly difficult, since time is limited and both PE and bleeding can be life-threatening. A multidisciplinary approach to discuss available options (ST, USAT, other catheter-directed or surgical options) is important. The present study affirms that, although USAT is thought to be associated with a lower incidence of bleeding, this type of thrombolysis still carries an important risk of bleeding and mortality. Further research involving patients with high-risk PE is warranted to clarify which patients will benefit most from USAT.

The main strength of the present study is the inclusion of all patients treated with USAT for high-risk PE in the Netherlands, with little loss to follow-up. Our study is the first case series of high-risk patients treated with USAT for PE. This illustrates how guidelines for high-risk PE are put to practice. Therefore, our results are generalizable to all hospitals treating patients according to current guidelines. In a setting of severe disease such as high-risk PE, results from case series are especially important to provide points of departure to guide patient selection.

Some limitations need to be addressed. First, our study population was small and heterogeneous. As only a small proportion of patients with PE has an indication for USAT, it is infrequently performed, which may limit individual experience. Treatment indications were heterogeneous, as decisions were made by the treating team of physicians. Other potential threats to internal validity are predominantly due to the retrospective design. Information bias could be introduced as data is extracted from medical records. Especially in acute situations, information on baseline characteristics might have been underreported. Moreover, even though hemodynamic stability was clearly defined, misclassification could still have occurred.

## Conclusion

Although guidelines advocate the use of USAT in those with high-risk PE and contraindications for thrombolytic treatment, especially in this population it is associated with a very high incidence of major bleeding and mortality. As the first case series of solely high-risk PE patients treated with USAT, this study adds important data on outcomes of this understudied population. Our results illustrate that at least caution is warranted in critically ill patients with high-risk PE when considering USAT. Further research in high-risk patients is essential to establish the place of USAT as treatment of PE.

## Abbreviations

AZN: St. Antonius Hospital, Nieuwegein
CI: Confidence interval
CTPA: Computed tomographic pulmonary angiography
DOAC: Direct oral anticoagulant
ECMO: Extracorporeal membrane oxygenation
EKOS: EkoSonic Endovascular system (EKOS Corporation; Bothell, WA, USA)
EMC: Erasmus Medical Center Rotterdam
ISTH: International Society on Thrombosis and Haemostasis
IQR: Interquartile range
LMWH: Low-molecular-weight heparin
PE: Pulmonary embolism
RV: Right ventricle
ST: Systemic thrombolysis
UMCU: University Medical Center Utrecht
USAT: Ultrasound-assisted catheter-directed thrombolysis
VCF: Vena cava filter
VKA: Vitamin K antagonist
VTE: Venous thromboembolism
VUMC: VU University Medical Center

## References

[1] Konstantinides SV, Torbicki A, Agnelli G (2014). Task force for the diagnosis and management of acute pulmonary embolism of the European Society of Cardiology (ESC). 2014 ESC guidelines on the diagnosis and management of acute pulmonary embolism. Eur Heart J.

[2] Kasper W, Konstantinides S, Geibel A (1997). Management strategies and determinants of outcome in acute pulmonary embolism: results of a multicenter registry. J Am Coll Cardiol.

[3] Agnelli G, Becattini C (2010). Acute pulmonary embolism. N Engl J Med.

[4] Meyer G, Vicaut E, Danays T (2014). PEITHO investigators. Fibrinolysis for patients with intermediate-risk pulmonary embolism. N Engl J Med.

[5] Goldhaber SZ, Haire WD, Feldstein ML (1993). Alteplase versus heparin in acute pulmonary embolism: randomised trial assessing right-ventricular function and pulmonary perfusion. Lancet.

[6] Dalla-Volta S, Palla A, Santolicandro A (1992). PAIMS 2: alteplase combined with heparin versus heparin in the treatment of acute pulmonary embolism. Plasminogen activator Italian multicenter study 2. J Am Coll Cardiol.

[7] Chatterjee S, Chakraborty A, Weinberg I (2014). Thrombolysis for pulmonary embolism and risk of all- cause mortality, major bleeding, and intracranial hemorrhage: a meta-analysis. JAMA.

[8] Stein PD, Matta F (2012). Thrombolytic therapy in unstable patients with acute pulmonary embolism: saves lives but underused. Am J Med.

[9] Goldhaber SZ, Visani L, De Rosa M (1999). Acute pulmonary embolism: clinical outcomes in the International Cooperative Pulmonary Embolism Registry (ICOPER). Lancet.

[10] Kline JA, Nordenholz KE, Courtney DM (2014). Treatment of submassive pulmonary embolism with tenecteplase or placebo: cardiopulmonary outcomes at three months (TOPCOAT): multicenter double-blind, placebo-controlled randomized trial. J Thromb Haemost.

[11] Kucher N, Rossi E, De Rosa M, Goldhaber SZ (2006). Massive pulmonary embolism. Circulation.

[12] Owens CA (2008). Ultrasound-enhanced thrombolysis: EKOS EndoWave infusion catheter system. Semin Interv Radiol.

[13] EkoSonic^®^ Endovascular system receives FDA clearance for the treatment of pulmonary embolism in the USA. Cath Lab Digest 2014. http://www.cathlabdigest.com/EkoSonic%C2%AE-Endovascular-System-receives-FDA-Clearance-treatment-pulmonary-embolism-USA. Accessed 28 June 2017.

[14] Kucher N, Boekstegers P, Müller OJ (2014). Randomized, controlled trial of ultrasound-assisted catheter-directed thrombolysis for acute intermediate-risk pulmonary embolism. Circulation.

[15] Piazza G, Hohlfelder B, Jaff MR (2015). SEATTLE II investigators. A prospective, single-arm, multicenter trial of ultrasound-facilitated, catheter-directed, low-dose fibrinolysis for acute massive and submassive pulmonary embolism: the SEATTLE II study. JACC Cardiovasc Interv.

[16] Bloomer TL, El-Hayek GE, McDaniel MC (2017). Safety of catheter-directed thrombolysis for massive and submassive pulmonary embolism: results of a multicenter registry and meta-analysis. Catheter Cardiovasc Interv.

[17] Patel N, Patel NJ, Agnihotri K (2015). Utilization of catheter-directed thrombolysis in pulmonary embolism and outcome difference between systemic thrombolysis and catheter-directed thrombolysis. Catheter Cardiovasc Interv.

[18] Lee KA, Cha A, Kumar MH, Rezayat C, Sales CM (2017). Catheter-directed, ultrasound-assisted thrombolysis is a safe and effective treatment for pulmonary embolism, even in high-risk patients. J Vasc Surg Venous Lymphat Disord.

[19] Liang NL, Avgerinos ED, Singh MJ, Makaroun MS, Chaer RA (2017). Systemic thrombolysis increases hemorrhagic stroke risk without survival benefit compared with catheter-directed intervention for the treatment of acute pulmonary embolism. J Vasc Surg Venous Lymphat Disord.

[20] Sag S, Nas OF, Kaderli AA (2016). Catheter-directed ultrasound-accelerated thrombolysis may be life-saving in patients with massive pulmonary embolism after failed systemic thrombolysis. J Thromb Thrombolysis.

[21] Fuller TJ, Paprzycki CM, Zubair MH, Hussain LR, Kuhn BA, Recht MH, Muck PE (2017). Initial experiences with endovascular management of submassive pulmonary embolism: is it safe?. Ann Vasc Surg.

[22] Engelberger RP, Kucher N (2014). Ultrasound-assisted thrombolysis for acute pulmonary embolism: a systematic review. Eur Heart J.

[23] Tapson VF, Sterling K, Jones N (2018). A randomized trial of the optimum duration of acoustic pulse thrombolysis procedure in acute intermediate-risk pulmonary embolism: the OPTALYSE PE trial. JACC Cardiovasc Interv.

[24] Kaymaz C, Akbal OY, Hakgor A (2018). A five-year, single-centre experience on ultrasound-assisted, catheter-directed thrombolysis in patients with pulmonary embolism at high risk and intermediate to high risk. EuroIntervention.

[25] Kaymaz C, Öztürk S, Akbal O (2017). Ultrasound-assisted catheter-directed thrombolysis in high-risk and intermediate-high-risk pulmonary embolism: results from a single-center cohort. Angiology.

[26] McCabe JM, Huang PH, Riedl L, Eisenhauer AC, Sobieszczyk P (2015). Usefulness and safety of ultrasound-assisted catheter-directed thrombolysis for submassive pulmonary emboli. Am J Cardiol.

[27] Bagla S, Smirniotopoulos JB, van Breda A, Sheridan MJ, Sterling KM (2015). Ultrasound-accelerated catheter-directed thrombolysis for acute submassive pulmonary embolism. J Vasc Interv Radiol.

[28] Schulman S, Kearon C (2005). Subcommittee on Control of Anticoagulation of the Scientific and Standardization Committee of the International Society on Thrombosis and Haemostasis. Definition of major bleeding in clinical investigations of antihemostatic medicinal products in non-surgical patients. J Thromb Haemost.

